# Anxiety and depression among cancer patients in Ethiopia: a systematic review and meta-analysis

**DOI:** 10.3389/fpsyt.2024.1341448

**Published:** 2024-02-22

**Authors:** Habtamu Geremew, Samuel Abdisa, Elyas Melaku Mazengia, Werkneh Melkie Tilahun, Aysheshim Belaineh Haimanot, Tigabu Kidie Tesfie, Anteneh Lamesgen Mneneh, Muluye Gebrie Mengie, Bekalu Endalew, Molla Yigzaw Birhanu, Lakew Asmare, Mulat Belay Simegn

**Affiliations:** ^1^ College of Health Science, Oda Bultum University, Chiro, Ethiopia; ^2^ Department of Midwifery, College of Health Science, Oda Bultum University, Chiro, Ethiopia; ^3^ Department of Public Health, College of Medicine and Health Science, Debre Markos University, Debre Markos, Ethiopia; ^4^ Department of Epidemiology and Biostatistics, Institute of Public Health, College of Medicine and Health Sciences, University of Gondar, Gondar, Ethiopia; ^5^ Department of Epidemiology and Biostatistics, School of Public Health, College of Medicine and Health Science, Wollo University, Dessie, Ethiopia

**Keywords:** anxiety, depression, prevalence, cancer patients, Ethiopia

## Abstract

**Introduction:**

Anxiety and depression are among the common comorbidities of people diagnosed with cancer. However, despite the progress in therapeutic options and outcomes, mental health care and support have lagged behind for cancer patients. Estimating the extent and determinants of mental health disorders among cancer patients is crucial to alert concerned bodies for action. In view of this, we aimed to determine the pooled prevalence and determinants of anxiety and depression among cancer patients in Ethiopia.

**Methods:**

Relevant literatures were searched on PubMed, African Journals Online, Hinari, Epistemonikos, Scopus, EMBASE, CINAHL, Cochrane Library, and Gray literature sources. Data were extracted into an Excel spreadsheet and analyzed using STATA 17 statistical software. The random effect model was used to summarize the pooled effect sizes with their respective 95% confidence intervals. The I^2^ statistics and Egger’s regression test in conjunction with the funnel plot were utilized to evaluate heterogeneity and publication bias among included studies respectively.

**Results:**

A total of 17 studies with 5,592 participants were considered in this review. The pooled prevalence of anxiety and depression among cancer patients in Ethiopia were 45.10% (95% CI: 36.74, 53.45) and 42.96% (95% CI: 34.98, 50.93), respectively. Primary and above education (OR= 0.76, 95% CI: 0.60, 0.97), poor social support (OR= 2.27, 95% CI: 1.29, 3.98), occupational status (OR= 0.59; 95% CI: 0.43, 0.82), advanced cancer stage (OR= 2.19, 95% CI: 1.38, 3.47), comorbid illness (OR= 1.67; 95% CI: 1.09, 2.58) and poor sleep quality (OR= 11.34, 95% CI: 6.47, 19.89) were significantly associated with depression. Whereas, advanced cancer stage (OR= 1.59, 95% CI: 1.15, 2.20) and poor sleep quality (OR= 12.56, 95% CI: 6.4 1, 24.62) were the factors associated with anxiety.

**Conclusion:**

This meta-analysis indicated that a substantial proportion of cancer patients suffer from anxiety and depression in Ethiopia. Educational status, occupational status, social support, cancer stage, comorbid illness and sleep quality were significantly associated with depression. Whereas, anxiety was predicted by cancer stage and sleep quality. Thus, the provision of comprehensive mental health support as a constituent of chronic cancer care is crucial to mitigate the impact and occurrence of anxiety and depression among cancer patients. Besides, families and the community should strengthen social support for cancer patients.

**Systematic Review Registration:**

https://www.crd.york.ac.uk/prospero/, identifier CRD42023468621.

## Introduction

In the recent world, non-communicable diseases have become common and cancer is among the leading causes of death worldwide accounting for 1 every 6 deaths (1). In 2018, World Health Organization (WHO) estimated that cancer caused about 18 million new cases and 9.6 million deaths worldwide (2). Cancer is known to cause immature death mainly if it reaches an advanced stage without diagnosis and treatment. In addition, cancer is related to disturbing the victim’s psychological health by causing common mental health disorders like depression and anxiety (3). The burden of cancer is far higher in developing countries due to different factors mainly socio-demographic and weak prevention and control methods. In Ethiopia, cancer accounts for about 5.8% of total national mortality, and the incidence of cancer is estimated to be at least 60960 cases, and a death rate of more than 44000 each year (4).

Being diagnosed with cancer may cause many complex feelings and lifestyle changes in people who are diagnosed with it, and many people feel to blame for getting cancer and suffer from psychological disorders like depression and anxiety (5, 6). Depression and anxiety are known to complicate the lives of patients with cancer than the general population. More than 300 million people are now living with depression, an increase of more than 18% between 2005 and 2015 in the general population (7), whereas approximately half of all patients with cancer have common psychological disorders such as anxiety and depression (8). Both depression and anxiety among patients with cancer are associated with complicating the outcome of the cancer treatment since they have to take medications for both cancer and depression or anxiety, lower adherence, extend hospitalization, reduce the quality of life, increase the risk of suicide and predict cancer advancement and mortality (9–13).

Anxiety and depression co-morbidity among cancer patients is common in underdeveloped settings (14). In Ethiopia, few studies reported the magnitude of anxiety and depression among cancer patients. However, they present inconsistent findings and failed to summarize the national figure. For instance, depression was found to affect 15.3% to 70.9% of respondents (15, 16), whereas anxiety was figured from 27.8% to 64.9% (17, 18). This demonstrates substantial variation among previous studies.

These common mental disorders are related to various factors that make them more common, as per different literature. Factors like age, education level, poor patient-provider interaction, social support, financial support, sleep quality, and stage of cancer were documented as significant predictors of anxiety and depression among cancer patients (6, 16, 19, 20). Nevertheless, their importance varies between studies.

Although it needs global attention, most of the studies regarding common mental disorders among cancer patients came from the developed world (21), while very limited studies addressed the subject in low-income countries like Ethiopia (6, 18, 22). Even those studies showed no stated national level of depression and anxiety among patients with cancer. In addition, there is no consistent finding regarding associated factors of depression and anxiety among those studies. Therefore, this study aimed to assess the pooled prevalence and determinants of depression and anxiety among cancer patients in Ethiopia.

## Materials and methods

### Protocol registration and reporting

The protocol of this systematic review is available in the international database of the Prospective Register of Systematic Reviews (PROSPERO) with registration number CRD42023468621. This review closely followed the Preferred Reporting Items for Systematic Reviews and Meta-Analysis (PRISMA) statement (23).

### Search strategy

We did a comprehensive literature search on PubMed, African Journals Online (AJOL), Hinari, Epistemonikos, Scopus, EMBASE, CINAHL, and Cochrane Library to retrieve relevant articles. In addition, other gray literature like Google Scholar and online repositories of Ethiopian universities were audited. The reference list of pertinent articles was also scrutinized to identify additional studies. Two independent reviewers (HG and MBS) searched the databases from September 5 to October 5, 2023. The following keywords were used for the search: Prevalence OR “Period Prevalence” OR “Point Prevalence” OR Magnitude OR Epidemiology) AND (“Associated factor*” OR Determinant* OR Factor* OR “Risk factor*”) AND (Depression OR “Depressive Symptoms” OR “Emotional Depression” OR Anxiety OR Angst OR Anxiousness OR “Social Anxiety” OR “Anxiety Disorders”) AND (Neoplasm* OR “Benign Neoplasm*” OR Cancer OR Malignancy OR “Malignant Neoplasm” OR “Malignant Neoplasms” OR Neoplasia OR Benign OR Tumor*) AND (Ethiopia).

### Eligibility criteria

All observational studies published in the English language and reported the prevalence and/or determinants of anxiety and/or depression among cancer patients in Ethiopia were included in this review. Nonetheless, clinical trials, duplicate studies, editorial letters, qualitative studies, and abstracts without full text were excluded from this analysis.

Population: Cancer patients

Setting: Studies conducted only in Ethiopia

Exposure: predictors of anxiety and depression. These are attributes of cancer patients that may increase or decrease their likelihood of developing anxiety and depression, such as educational status, social support, cancer stage, sleep quality, and so forth.

Outcome: anxiety and depression

### Outcome of interest

Our primary outcome of interest was the magnitude of anxiety and depression among cancer patients in Ethiopia. In this analysis, the hospital anxiety and depression scale (HADS) with a cut-off point ≥ 8, and the patient health questionnaire (PHQ) with a cut-off point ≥ 10, were used to diagnose anxiety and depression among cancer patients (15, 22). Secondly, we also sought to identify determinants of anxiety and depression among cancer patients in Ethiopia.

### Study selection, quality assessment, and data extraction

Studies were assessed according to their titles and abstracts after duplicates were eliminated. For studies found to be suitable by title and abstract, a full-text review against pre-defined inclusion/exclusion criteria was done to determine potential articles to be included in this analysis. Reference management software (Endnote version X7.2) was used to combine database search results, manage the citation process, and remove duplicate entries.

Quality of included studies was assessed using the Joanna Brigg’s Institute (JBI) quality assessment checklist (24), (**Supplementary File 1**). Two authors of the review (HG and MBS) independently evaluated the quality of each study and inconsistencies were resolved by involving a third author (SA).

The data were extracted into to Excel spreadsheet using a standard data extraction form that was developed considering the JBI guide for data extraction and synthesis (25). Two independent authors extracted records of primary studies and then cross-checked one another. Extracted data include last name of the first author and year of publication, study area, study participants (all cancer patients or specific cancer type), methods employed (hospital anxiety and depression scale or patient health questionnaire), outcome reported (anxiety and/or depression), sample size and prevalence of anxiety and/or depression. Besides, data about determinants of anxiety and/or depression were also abstracted in a two-by-two table format.

### Data processing and analysis

Data were extracted using the Excel spreadsheet and then imported to STATA version 17 software for further statistical analysis. The prevalence and its standard error of each primary study were considered to calculate the pooled prevalence estimates. The random effects model was used to pool the prevalence of anxiety and depression, and estimates were presented by forest plots. Heterogeneity between included studies was assessed using the I^2^ statistics and it was regarded as high, moderate, or low when I^2^ test statistics results were 75%, 50%, and 25% respectively (26). Eggers regression test in conjunction with funnel plot was used to evaluate publication bias and a p-value of less than 0.05 was considered to declare the presence of publication bias (27). In light of the high heterogeneity among included studies, subgroup analysis was conducted by study area and method of diagnosis to further explore it. Besides, a leave-one-out sensitivity analysis was done to evaluate the impact of each study on the overall estimate.

## Results

### Study selection

**Figure 1:**
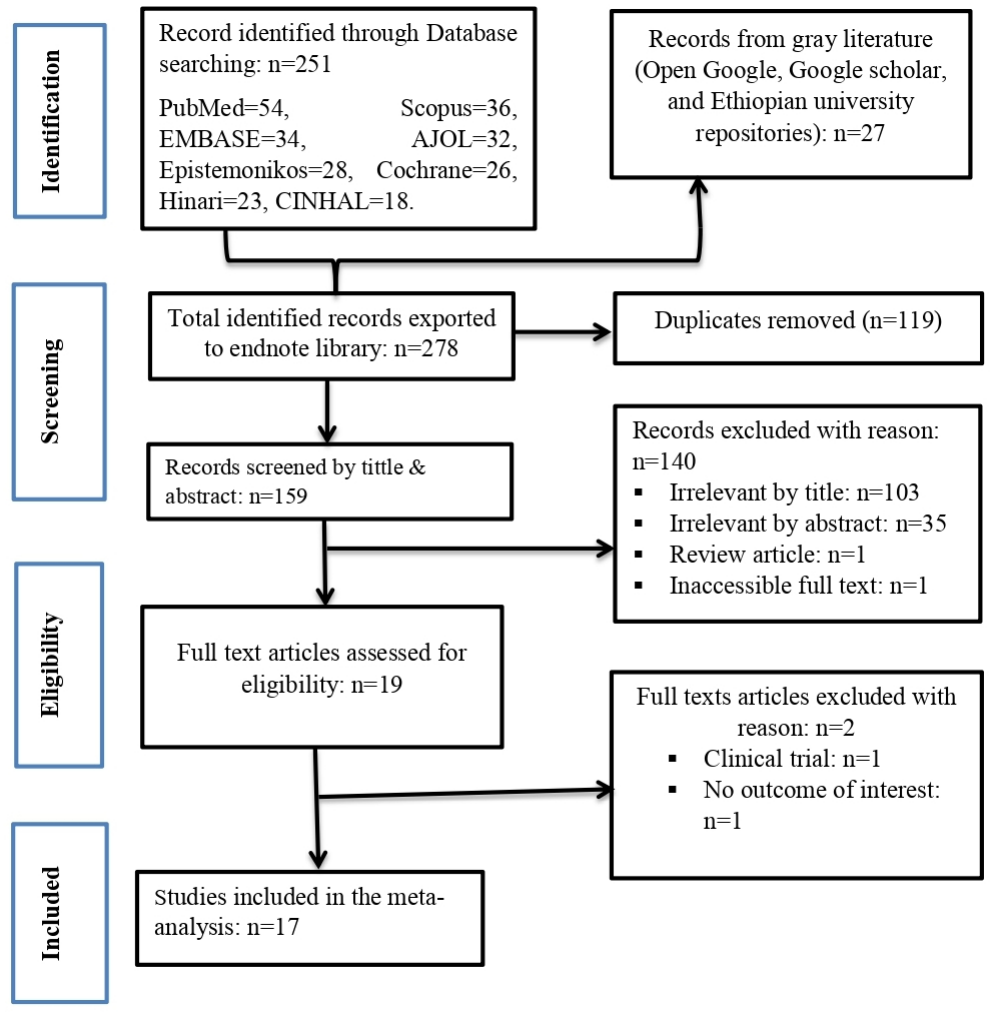
PRISMA flow chart for articles screened and included.

The combined literature search gave us a total of 278 studies. After the removal of 119 duplicate studies, 159 studies were screened by reviewing their title and abstract of which 140 articles were irrelevant. The full texts of 19 studies were assessed and 17 studies were deemed suitable for inclusion in this meta-analysis (**Figure 1**).

### Characteristics of included studies

Among the 17 studies included in this analysis, one study reported anxiety alone (28), five studies reported depression alone (15, 16, 29–31), while the remaining 11 studies reported both depression and anxiety (6, 17, 18, 20, 22, 32–37). Correspondingly, the magnitudes of anxiety and depression were determined by pooling findings of 12 (6, 17, 18, 20, 22, 28, 32–37) and 16 (6, 15–18, 20, 22, 29–37) studies respectively. All included studies utilized institutional based cross-sectional study design with a sample size ranging from 77 (34), to 428 (31). Two studies included breast cancer patients only (31, 33), while others included all malignancies (6, 15–18, 20, 22, 28–30, 32, 34–37). All included primary studies involved cancer patients who were receiving care. Regarding the methods of diagnosis, 12 studies utilized the hospital anxiety and depression scale (6, 17, 18, 20, 22, 28, 32–37), while five studies used the patient health questionnaire (15, 16, 29–31). Nine of the studies were conducted in the national capital, Addis Ababa (15, 18, 22, 28–33), whereas eight of the included studies were conducted in different regions of the country (6, 16, 17, 20, 34–37) (**Table 1**).

**Table 1:** Characteristics of studies included in the meta-analysis.

Author, publication year	Study area	Participants	Methods employed	Outcomes reported	Sample size	Risk of bias
Abraham et al, 2022 (22)	Addis Ababa	All cancer patients	HADS	Anxiety and depression	423	Low risk
Alemayehu et al, 2018 (29)	Addis Ababa	All cancer patients	PHQ	Depression	390	Low risk
Atinafu et al, 2022 (18)	Addis Ababa	All cancer patients	HADS	Anxiety and depression	171	Low risk
Ayalew et al, 2022 (6)	SNNP*	All cancer patients	HADS	Anxiety and depression	415	Low risk
Baraki et al, 2020 (16)	Amhara	All cancer patients	PHQ	Depression	302	Low risk
Belay et al, 2022 (33)	Addis Ababa	Breast cancer patients	HADS	Anxiety and depression	333	Low risk
Belete et al, 2022 (30)	Addis Ababa	All cancer patients	PHQ	Depression	420	Low risk
Berihun et al, 2017 (34)	Amhara	All cancer patients	HADS	Anxiety and depression	77	Low risk
Endeshaw et al, A 2022 (20)	Amhara	All cancer patients	HADS	Anxiety and depression	392	Low risk
Wondimagegnehu et al, 2019 (31)	Addis Ababa	Breast cancer patients	PHQ	Depression	428	Low risk
Wurjine et al, 2020 (28)	Addis Ababa	All cancer patients	HADS	Anxiety	220	Low risk
Abebe et al, 2023 (32)	Addis Ababa	All cancer patients	HADS	Anxiety and depression	264	Low risk
Degefa et al, 2020 (15)	Addis Ababa	All cancer patients	PHQ	Depression	163	Low risk
Endeshaw et al, B 2022 (35)	Amhara	All cancer patients	HADS	Anxiety and depression	410	Low risk
Hagezom et al, 2021 (17)	Tigray	All cancer patients	HADS	Anxiety and depression	410	Low risk
Molla et al, 2022 (36)	Amhara	All cancer patients	HADS	Anxiety and depression	416	Low risk
Nigussie et al, 2023 (37)	Harari	All cancer patients	HADS	Anxiety and depression	358	Low risk

HADS, Hospital anxiety and depression scale; PHQ, Patient health questionnaire; SNNPR*, Southern Nations, Nationalities and Peoples of Ethiopia.

### Prevalence of anxiety and depression

**Figure 2:**
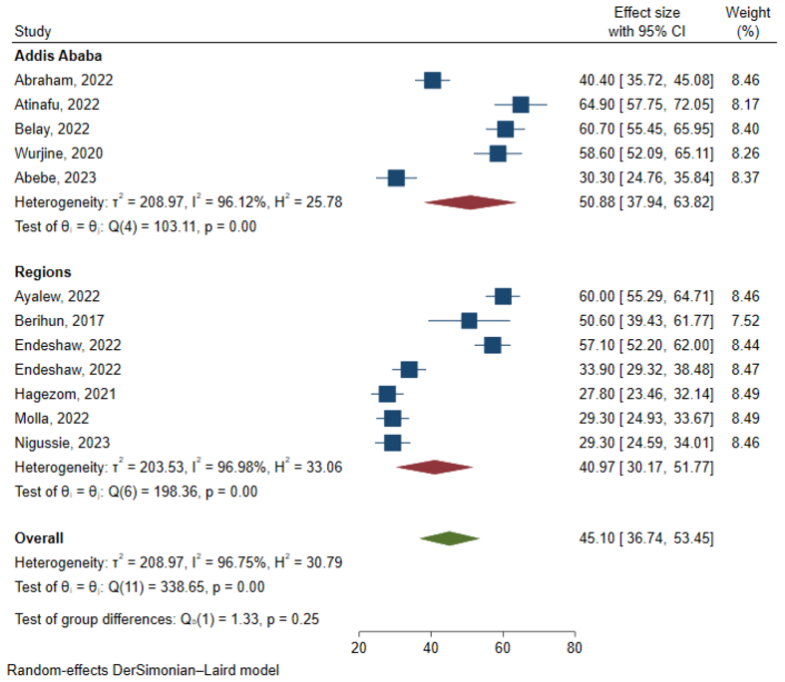
Forest plot for prevalence of anxiety among cancer patients in Ethiopia.

**Figure 3:**
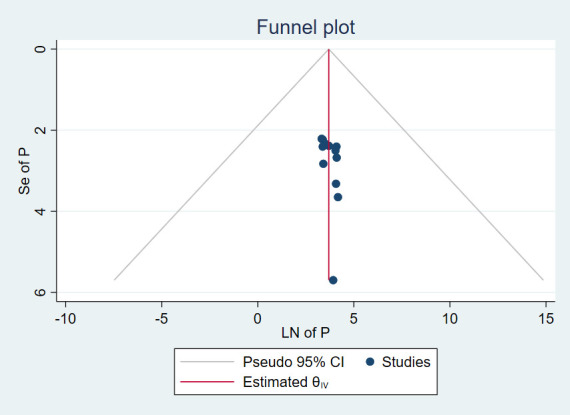
Funnel plot, evaluating existence of publication bias for prevalence of anxiety among cancer patients in Ethiopia.

The results of 12 studies involving 3,889 respondents indicated that the overall pooled prevalence of anxiety among cancer patients in Ethiopia was 45.10% (95% CI: 36.74, 53.45) (**Figure 2**). Egger’s regression test indicated that there was no evidence of publication bias (p = 0.173). The symmetrical funnel plot further strengthens this finding (**Figure 3**).

**Figure 4:**
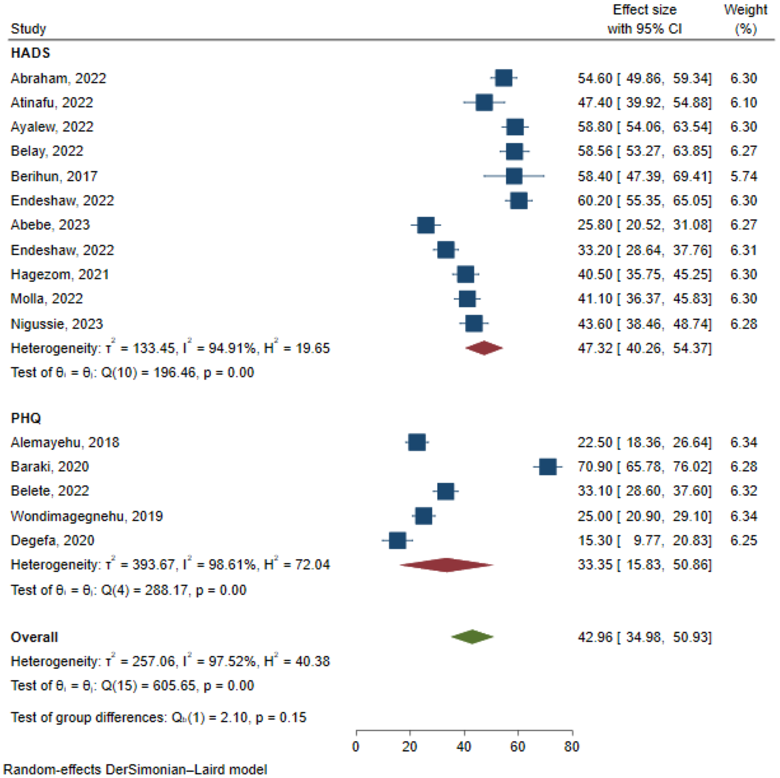
Forest plot for prevalence of depression among cancer patients in Ethiopia.

**Figure 5:**
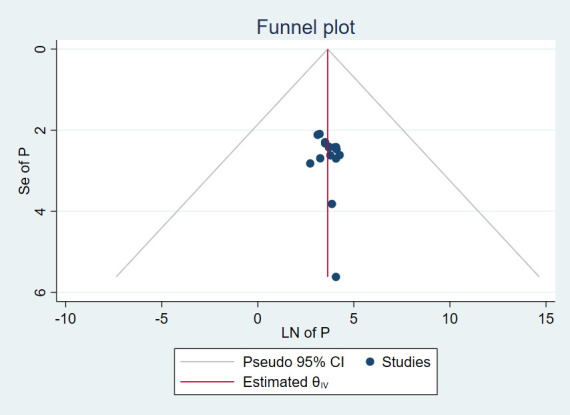
Funnel plot, evaluating the existence of publication bias for prevalence of depression among cancer patients in Ethiopia.

After pooling the results of 16 studies with 5,372 participants, the estimated overall prevalence of depression among cancer patients in Ethiopia was 42.96% (95% CI: 34.98, 50.93) (**Figure 4**). Findings of Egger’s regression test (p = 0.237) and funnel plot (**Figure 5**) indicated no evidence of publication bias.

### Heterogeneity and publication bias

**Figure 6:**
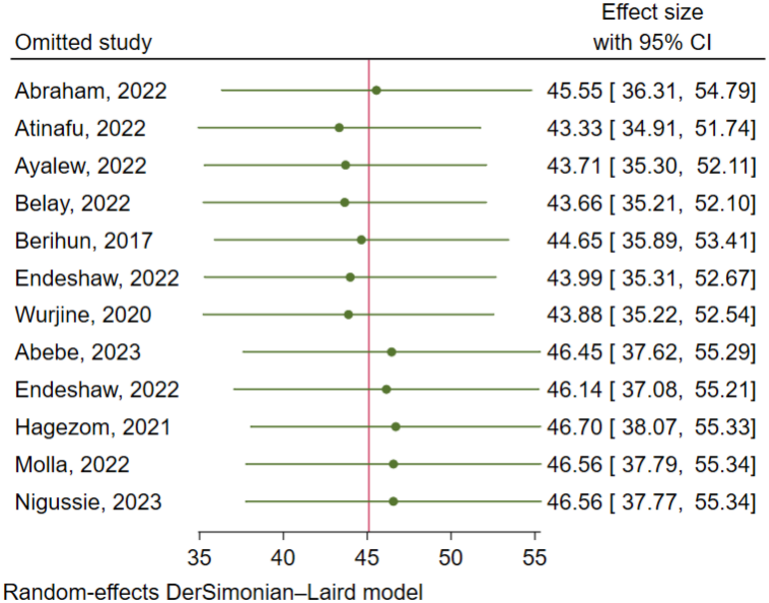
Sensitivity analysis for prevalence of anxiety among cancer patients in Ethiopia.

**Figure 7:**
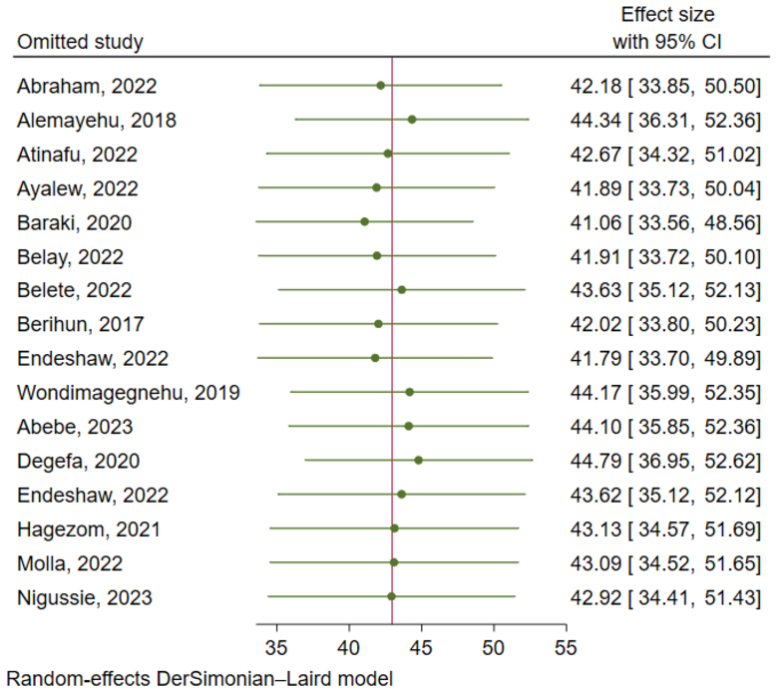
Sensitivity analysis for prevalence of depression among cancer patients in Ethiopia.

There was a significant level of heterogeneity among included studies for both the anxiety and depression analysis with the Cochrane I^2^ values of 96.75% and 97.52% respectively. To account for this substantial heterogeneity, the random effect model (DerSimonian-Laird method) was used to determine the pooled estimates. In both cases, there was no evidence of publication bias as depicted by a symmetrical funnel plot and insignificant Egger’s regression test. In addition, a leave-one-out sensitivity analysis was employed to investigate the influence of each study on the overall estimate. As a result, the pooled estimates of anxiety (**Figure 6**) and depression (**Figure 7**) among cancer patients in Ethiopia were steady and reliable when analyzed by omitting one study at a time.

### Factors associated with anxiety

Data about five different variables (marital status, educational status, cancer stage, sleep quality, and social support) were extracted in a two-by-two table and analyzed separately. As a result, the pooled effect size of two variables (cancer stage and sleep quality) showed a significant association with anxiety. Accordingly, the likelihood of experiencing anxiety symptoms was 59% higher among individuals with advanced-stage cancer than individuals with non-advanced cancer (OR= 1.59; 95% CI: 1.15, 2.20). Similarly, cancer patients who had poor sleep quality were 12.56 (95% CI: 6.41, 24.62) times more likely to experience anxiety than their counterparts (**Table 2**). On the other hand, pooling the effect size of some variables deemed meaningless because of the marked variation in categories between the primary studies. For instance, six studies investigated the association between age and occurrence of anxiety; five of them found no significant association between age and anxiety, even if some of the studies treated age as continuous variable while others categorized it (6, 20, 28, 33, 34). In contrast, one study showed a statistically significant association between age and anxiety, where older patients had higher risk of anxiety (18). But, combining the effect size of those studies is misleading because of the heterogeneous categories between studies. Besides, some reports treated age as continuous variable while others categorized it.

**Table 2:** Summary estimate of OR for factors associated with anxiety among cancer patients in Ethiopia.

Variables	Exposed	Comparator	Included studies	OR (95% CI)	I^2^
Cancer stage	Advanced	Not advanced	2	1.59 (1.15, 2.20)	0.0%
Sleep quality	Poor	Good	2	12.56 (6.4 1, 24.62)	28.3%%

### Factors associated with depression

For predictors of depression, data about all variables that were reported to have a significant association in one of the included studies and also recorded in at least one other study (with or without significant association) were extracted in a two-by-two table and analyzed separately. Subsequently, data about the association between depression and nine independent variables (sex, educational status, occupational status, stage of cancer, anatomical site of cancer, sleep quality, social support, nutritional status and presence of other comorbid illness) were extracted into excel spreadsheet and analyzed separately. The pooled effects of six variables (educational status, occupational status, stage of cancer, sleep quality, social support and comorbid illness) indicated a significant association with depression among cancer patients in Ethiopia. Correspondingly, the risk of depression was 24% lower among cancer patients who had attended formal education (primary or above) as compared to those who had not attended formal education (OR= 0.76; 95% CI: 0.60, 0.97). Unemployed cancer patients had 41% higher risk of depression than employed ones (OR= 0.59; 95% CI: 0.43, 0.82). Patients who had advanced-stage cancer were more than two times more likely to suffer from depression than their counterparts (OR= 2.19; 95% CI: 1.38, 3.47). Similarly, the odds of depression were about 11 times higher among cancer patients who had poor sleep quality than those who had good sleep quality (OR= 11.34; 95% CI: 6.47, 19.89). Cancer patients who had other comorbid illness were 1.67 times more likely to suffer from depression than cancer patients who did not had comorbid illness (OR= 1.67; 95% CI: 1.09, 2.58). Lastly, we found that cancer patients who received poor social support had a higher risk of depression as compared to those who received strong social support (OR= 2.27; 95% CI: 1.29, 3.98) (**Table 3**).

**Table 3:** Summary estimate of OR for factors associated with depression among cancer patients in Ethiopia.

Variables	Exposed	Comparator	Included studies	OR (95% CI)	I^2^
Education	Primary and above	No formal education	4	0.76 (0.60, 0.97)	0.6%
Cancer stage	Advanced	Not advanced	3	2.19 (1.38, 3.47)	63.1%
Sleep quality	Poor	Good	2	11.34 (6.47, 19.89)	0.0%
Social support	Good	Moderate	3	1.00 (0.68, 1.47)	14.8%
		Poor	3	2.27 (1.29, 3.98)	56.3%
Occupational status	Employed	Unemployed	3	0.59 (0.43, 0.82)	0.0%
Comorbid illness	Yes	No	2	1.67 (1.09, 2.58)	0.0%

However, some variables like age were differently categorized across studies, thus making effect size combination inappropriate. Generally, nine studies assessed the association between age and depression. Of these, five studies indicated that age was not significantly associated with depression (16, 18, 20, 29, 34), whereas four studies reported a statistically significant association between age and depression, out of which three of them showed higher risk of depression among younger patients (22, 31, 33), while one study showed higher risk of depression among older cancer patients (6). This variation between studies might be due to the difference in age categorization and/or sample size across the primary studies.

## Discussion

In spite of the development in therapeutic options and outcomes, mental health care and support have lagged behind for cancer patients (38). The problem is more prominent in underdeveloped settings where there are prevalent cases, adding to the poor resources needed to implement comprehensive care for people with cancer (39). Estimating the extent and determinants of mental health disorders among cancer patients is crucial to alert concerned bodies for action. In view of this, this study was conducted to estimate the magnitude of anxiety and depression among cancer patients in Ethiopia.

In this review, the pooled prevalence of anxiety among cancer patients in Ethiopia was 45.10% (95% CI: 36.74, 53.45). The estimate is in line with a recent meta-analysis by Hashemi et.al, 41.9% (40). This finding is also congruent with recent meta-analysis conducted in Iran 40.9% (41), and China 38% (42). However, our estimate was quite high when compared to a meta-analysis of studies from developed settings, 15.09% (43). This variation could be explained in part by the large difference in socioeconomic status and healthcare quality between high-income and low-income countries (39).

The pooled estimate of depression among cancer patients in Ethiopia was 42.96% (95% CI: 34.98, 50.93). Our depression estimate was also in line with other meta-analyses from China 38% (42), and Iran 50.1% (41). Nevertheless, the depression prevalence estimate in this study was higher than in a previous meta-analysis conducted in Low- and Lower-Middle–Income Countries 21% (39). The possible explanation for this variation might be due to the difference in methods employed to assess depression (44). The differences in study design, study settings, and sample size could also be another elucidation for the observed discrepancy.

The present study also identified factors associated with depression and anxiety. Educational status, social support, stage of cancer, and sleep quality were found to have a significant association with depression. Whereas, stage of cancer and sleep quality were significant determinants of anxiety. Accordingly, stage of cancer and sleep quality were significantly associated with both anxiety and depression. The odds of depression were more than two times higher among patients with advanced-stage cancer than their counterparts. Similarly, the risk of anxiety symptoms was 59% higher among individuals with advanced stage of cancer than individuals with non-advanced cancer. These findings are supported by earlier reports (45, 46). This might be due to the physical impediment and disability to perform activities of daily living with the advancement of cancer (47). The odds of anxiety and depression were also higher among cancer patients who had poor sleep quality as compared to their counterparts. This might be due to the cognitive deterioration and other sleep-induced physiological changes associated with poor sleep (48). Hence, implies the importance of including regular and good sleep pattern counseling in chronic cancer care (49).

This study showed that cancer patients who had attended formal (primary or above) education had a 24% lower risk of depression as compared to those who had not attended formal education. This association was also demonstrated by previous studies conducted in Germany (19), and India (50). This might be due to the lack of knowledge and understanding about the disease they are diagnosed with among patients without formal education (51). Unemployed cancer patients had a 41% higher risk of depression than employed ones. This association was also documented in a previous meta-analysis (52). The possible explanation for this association might be that unemployed individuals frequently have unhealthy lifestyle and habits, which in turn raise their risk of depression (53).

In the present study, cancer patients with poor social support had more than twofold risk of depression than those with good social support. This association was also documented by previous studies (46, 54), and underlines the key role of social support in mitigating the negative impact of cancer diagnosis and treatment through the belonging and care they receive from others (55). Similarly, cancer patients who had other comorbid illness were more likely to suffer from depression than their counterparts. This might be due to the increased therapeutic burden and physical deterioration associated with other comorbid illness (56).

This analysis is not without limitations. Firstly, some important variables were not considered in this analysis due to differences in categorization across included primary studies. Secondly, studies published only in the English language were included in this review. Finally, all studies included in this analysis employed cross-sectional design, hence temporal associations are difficult to infer. In addition, all participants included in primary studies were cancer patients who were receiving care, hence the rate of anxiety and depression cannot be depicted by treatment status of cancer patients.

## Conclusion

This meta-analysis found that a substantial proportion of cancer patients suffer from anxiety and depression in Ethiopia. Educational status, occupational status, social support, cancer stage, comorbid illness and sleep quality were significantly associated with depression. Whereas, anxiety was predicted by cancer stage and sleep quality. Thus, the provision of comprehensive mental health support as a constituent of chronic cancer care is crucial to mitigate the impact and occurrence of anxiety and depression among cancer patients. Besides, families and the community should strengthen social support for cancer patients.

## Data availability statement

The original contributions presented in the study are included in the article/**Supplementary Material**. Further inquiries can be directed to the corresponding author.

## Author contributions

HG: Conceptualization, Data curation, Formal Analysis, Investigation, Methodology, Project administration, Resources, Software, Supervision, Validation, Visualization, Writing – original draft, Writing – review & editing. SA: Data curation, Investigation, Methodology, Project administration, Resources, Software, Supervision, Validation, Visualization, Writing – original draft, Writing – review & editing. EM: Data curation, Investigation, Methodology, Project administration, Resources, Validation, Visualization, Writing – review & editing. WT: Data curation, Investigation, Methodology, Project administration, Resources, Validation, Visualization, Writing – review & editing. AH: Data curation, Investigation, Methodology, Project administration, Resources, Validation, Visualization, Writing – review & editing. TT: Data curation, Investigation, Methodology, Project administration, Resources, Validation, Visualization, Writing – review & editing. AM: Data curation, Investigation, Methodology, Project administration, Resources, Validation, Visualization, Writing – review & editing. MM: Data curation, Investigation, Methodology, Project administration, Resources, Validation, Visualization, Writing – review & editing. BE: Data curation, Investigation, Methodology, Project administration, Resources, Validation, Visualization, Writing – review & editing. MB: Data curation, Investigation, Methodology, Project administration, Resources, Validation, Visualization, Writing – review & editing. LA: Data curation, Investigation, Methodology, Project administration, Resources, Validation, Visualization, Writing – review & editing. MS: Data curation, Formal Analysis, Investigation, Methodology, Project administration, Resources, Software, Supervision, Visualization, Writing – original draft, Writing – review & editing.
