# Supplementary material for: Anxiety and depression among cancer patients in Ethiopia: a systematic review and meta-analysis
Source: Front Psychiatry. 2024 Feb 22;15:1341448. doi: 10.3389/fpsyt.2024.1341448 (PMC10917887; doi:10.3389/fpsyt.2024.1341448)
Supplement: Supplementary file 1 [file Table_1.docx]

Supporting file 1, Risk of bias assessment for included studies

| First author and year of publication | **JBI Critical Appraisal Checklist** | | | | | | | | | Total score in %, risk of bias |
| --- | --- | --- | --- | --- | --- | --- | --- | --- | --- | --- |
|  | Was the sample frame appropriate to address the target population? | Were study participants sampled in an appropriate way? | Was the sample size adequate? | Were the study subjects and the setting described in detail? | Was the data analysis conducted with sufficient coverage of the identified sample? | Were valid methods used for the identification of the condition? | Was the condition measured in a standard, reliable way for all participants? | Was there appropriate statistical analysis? | Was the response rate adequate, & if not, was the low response rate managed appropriately? |  |
| Abraham et al, 2022 [22] | Yes | Yes | Yes | Yes | Yes | Yes | Yes | Yes | Yes | 100% ,  Low risk |
| Alemayehu et al, 2018 [29] | Yes | Yes | Yes | Yes | Yes | Yes | Unclear | Yes | Yes | 88.9%,  Low risk |
| Atinafu et al, 2022 [18] | Yes | Yes | Unclear | Yes | No | Yes | Yes | Yes | No | 66.7%, Low risk |
| Ayalew et al, 2022 [6] | Yes | Yes | Yes | Yes | Yes | Yes | Yes | Yes | Yes | 100%, Low risk |
| Baraki et al, 2020 [16] | Yes | Yes | Yes | Yes | Yes | Yes | Yes | Yes | Yes | 100%, Low risk |
| Belay et al, 2022 [33] | Yes | Unclear | Yes | Yes | Yes | Yes | Unclear | Yes | Yes | 77.8%, Low risk |
| Belete et al, 2022 [30] | Yes | Yes | Yes | Yes | Yes | Yes | Yes | Yes | Yes | 100%, Low risk |
| Berihun et al, 2017 [34] | Yes | Yes | Unclear | Yes | Unclear | Yes | Yes | No | Unclear | 55.6%, Low risk |
| Endeshaw et al, A 2022 [20] | Yes | Yes | Yes | Yes | Yes | Yes | Yes | Yes | Yes | 100%, Low risk |
| Wondimagegnehu et al, 2019 [31] | Yes | Unclear | Yes | Yes | Yes | Yes | Yes | Yes | Yes | 88.9%, Low risk |
| Wurjine et al, 2020 [28] | Unclear | No | Yes | Yes | Yes | Yes | Yes | Yes | Yes | 77.8%, Low risk |
| Abebe et al, 2023 [32] | Yes | Yes | Yes | Yes | Yes | Yes | Yes | Yes | Yes | 100%, Low risk |
| Degefa et al, 2020 [15] | Yes | Unclear | Yes | Yes | Yes | Yes | Yes | Yes | Yes | 88.9%,  Low risk |
| Endeshaw et al, B 2022 [35] | Yes | Yes | Yes | Yes | Yes | Yes | Yes | Yes | Yes | 100%, Low risk |
| Hagezom et al, 2021 [17] | Yes | Yes | Yes | Yes | Yes | Yes | Yes | Yes | Yes | 100%, Low risk |
| Molla et al, 2022 [36] | Yes | Yes | Yes | Yes | Yes | Yes | Yes | Yes | Yes | 100%, Low risk |
| Nigussie et al, 2023 [37] | Yes | Yes | Yes | Yes | Yes | Yes | Yes | Yes | Yes | 100%, Low risk |
